# pH-Responsive Chitosan/Alginate Polyelectrolyte Complexes on Electrospun PLGA Nanofibers for Controlled Drug Release

**DOI:** 10.3390/nano11071850

**Published:** 2021-07-17

**Authors:** Jean Schoeller, Fabian Itel, Karin Wuertz-Kozak, Sandra Gaiser, Nicolas Luisier, Dirk Hegemann, Stephen J. Ferguson, Giuseppino Fortunato, René M. Rossi

**Affiliations:** 1 Empa, Swiss Federal Laboratories for Materials Science and Technology, Laboratory for Biomimetic Membranes and Textiles, 9014 St. Gallen, Switzerland; jean.schoeller@empa.ch (J.S.); fabian.itel@empa.ch (F.I.); nicolas.luisier@gmail.com (N.L.); giuseppino.fortunato@empa.ch (G.F.); 2 Institute for Biomechanics, ETH Zürich, 8093 Zürich, Switzerland; sferguson@ethz.ch; 3 Schoen Clinic Munich Harlaching, Spine Center, Academic Teaching Hospital and Spine Research Institute of the Paracelsus Medical University Salzburg (AU), 81547 Munich, Germany; kwbme@rit.edu; 4 Department of Biomedical Engineering, Rochester Institute of Technology (RIT), Rochester, NY 14607, USA; 5 Empa, Swiss Federal Laboratories for Materials Science and Technology, Laboratory for Advanced Fibers, 9014 St. Gallen, Switzerland; sandra.gaiser@empa.ch (S.G.); dirk.hegemann@empa.ch (D.H.)

**Keywords:** electrospinning, pH-responsive, drug delivery, surface functionalization, layer-by-layer, natural polymers, surface charge, oral delivery

## Abstract

The surface functionalization of electrospun nanofibers allows for the introduction of additional functionalities while at the same time retaining the membrane properties of high porosity and surface-to-volume ratio. In this work, we sequentially deposited layers of chitosan and alginate to form a polyelectrolyte complex via layer-by-layer assembly on PLGA nanofibers to introduce pH-responsiveness for the controlled release of ibuprofen. The deposition of the polysaccharides on the surface of the fibers was revealed using spectroscopy techniques and ζ-potential measurements. The presence of polycationic chitosan resulted in a positive surface charge (16.2 ± 4.2 mV, pH 3.0) directly regulating the interactions between a model drug (ibuprofen) loaded within the polyelectrolyte complex and the layer-by-layer coating. The release of ibuprofen was slowed down in acidic pH (1.0) compared to neutral pH as a result of the interactions between the drug and the coating. The provided mesh acts as a promising candidate for the design of drug delivery systems required to bypass the acidic environment of the digestive tract.

## 1. Introduction

Electrospinning is a powerful technique to produce non-woven fibrous meshes from polymer solutions or polymer melts. Electrospun nanofibers present several advantages such as high surface-to-volume (*S*/*V*) ratio and high porosity. Furthermore, the design of responsive electrospun nanofibers has drawn significant attention in the past decade due to their ability to undergo physical or chemical changes when subjected to different stimuli. Such stimuli include pH, temperature, light, and electrical and magnetic fields [1,2,3,4]. “Smart” responsive nanofibers have found applications for biomedical purposes as scaffolds for tissue engineering, drug delivery systems, biosensors, and wound dressings [5,6,7,8,9,10,11,12,13,14]. When considering drug delivery, pH responsiveness has been widely investigated, especially for the design of systems providing a targeted and controlled release of active pharmaceutical ingredients. Indeed, pH-responsive systems can overcome biological barriers by restricting the release to certain pH values and therefore preventing systemic exposure of the drug to the patient due to the different pH environments within the human body. For the development of responsive drug delivery systems, nanofibers have shown great potential compared to other planar substrates. Mainly due to their high permeability and porosity, nanofibers have proven to provide shorter response times to environmental changes and therefore better control over the drug release [15].

**Figure 1:**
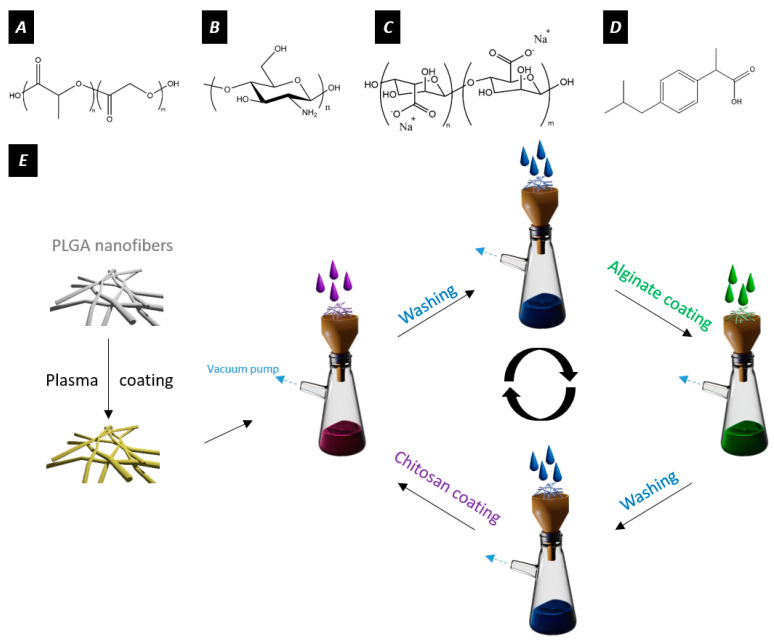
Chemical structures of (**A**) PLGA, (**B**) chitosan, (**C**) alginate, and (**D**) ibuprofen. (**E**) Summary of the LbL process for functionalizing the fibers. In this study, alginate and chitosan layers were alternatively deposited on the surface of plasma-coated electrospun nanofibers. The alginate and chitosan solutions are depicted in green and purple, respectively.

When designing drug delivery platforms, one must consider the biocompatibility and biodegradability of the materials used, as such systems are developed to integrate different cellular environments and thus are likely to degrade over time. Poly(lactic-*co*-glycolic acid) (PLGA, Figure 1A) has proven to be a suitable material due to its well-known biocompatibility and biodegradability [16]. Natural polymers have also emerged as excellent candidates for drug delivery systems. For this purpose, alginate (ALG, Figure 1C), a water-soluble anionic polysaccharide, is used for numerous biomedical applications thanks to its biocompatibility, biodegradability, and non-toxicity [17,18,19]. Furthermore, chitosan (CHI, Figure 1B), an abundant linear polysaccharide of deacetylated β-1,4-D-glucosamine, has been widely used in the past decade for its unique properties [20]. In fact, chitosan is one of the only natural cationic polymers containing primary amines in its polymeric backbone. Therefore, the assembly of CHI in solution is highly dependent upon protonation of the amino group (pKa ≈ 6.1). Once the amino function gets protonated, the polymeric backbone of chitosan becomes charged, leading to charge repulsion and swelling [21,22,23,24]. CHI and ALG form polyelectrolyte complexes in solution or on surfaces due to their oppositely charged polymeric backbones [25]. Such polyelectrolyte complexes are known to provide better mechanical and chemical stability than the isolated compounds while retaining their pH-sensitive behavior [17,26,27].

Electrospinning has been widely studied in the past decades, with a recent focus on the potential of surface modifications to achieve more sophisticated nanofibrous systems [28,29]. This can be achieved via crosslinking, coating, or chemical modifications of electrospun nanofibers [7,28]. For polymer coatings, layer-by-layer assembly (LbL) has proven to be a robust, versatile, and reproducible method for the grafting of polymers onto surfaces using electrostatic interactions [30,31,32,33,34]. LbL exploits the charges of the polymeric backbone of polymers to favor interactions with an oppositely charged polymer in order to deposit a thin layer over the surface. Nanofibers show great potential for this technique due to their high surface-to-volume ratio. Several groups have studied the feasibility of LbL on electrospun nanofibers. For example, using LbL-inspired techniques, Lin et al. coated poly(lactic acid) electrospun nanofibers with chitosan [29]. The coated fibers exhibited a better crystallization of calcium phosphate, thus making the system a potential candidate for bone tissue engineering. Moreover, Croisier et al. have grafted CHI onto the surface of electrospun poly(ε-caprolactone) fibers to provide anti-bacterial properties to the overall system [35]. In another study, Wang et al. developed a vascular graft based on poly-ε-caprolactone coated with polyethylene imine and heparin for the sustained delivery of epigallocatechin gallate [32]. In addition, Deng et al. have deposited CHI/ALG polyelectrolyte complexes on the surface of cellulose nanofibers [36]. The fibers exhibited better cell growth than the pristine fibers. Although several studies have revealed the capacity of electrospun nanofibers to act as substrates for the deposition of polymeric layers via LbL, no reports were made about the evaluation of such systems for pH-sensitive drug release. Nevertheless, this technique can act as a versatile approach to tailor loading and release kinetics.

Here, we investigate the ability of polyelectrolyte-coated PLGA nanofibers to release ibuprofen (IBU, Figure 1D) in response to environmental changes in pH. After incorporating negative charges via plasma coating onto the surface of PLGA nanofibers, we sequentially deposited the desired number of layers of CHI and ALG using LbL. Then, the specimens were characterized to study the deposition of the polymeric layers onto the surface of the fibers. Ibuprofen served as a model drug and was loaded onto the coating. The influence of the number of layers on the loading efficiency as well as the release kinetics with alterations in pH was highlighted. Figure 1E summarizes the process used for LbL coatings. The choice of material properties, combining oppositely charged polymers, control over layer thickness, and thus diffusion properties, as well as pH sensitive units make this approach highly versatile for the controlled loading and delivery of drugs. Clearly distinguished drug release kinetics at neutral pH (7.4) compared to an acidic pH (1.0) allow for tuned drug delivery in response to the environment. Thereby, drug-loaded electrospun membranes are protected from drug release in the acidic environment of the stomach and reach their target of higher environmental pH without previous loss and reduced drug concentration. In the future, this will help to bypass side effects with drug release at non-specified sites and help to fine-tune the host and target specific therapies. In this study, we show the applicability of LbL coatings on the surface of electrospun nanofibers for the design of pH-sensitive drug delivery systems.

## 2. Materials and Methods

*Materials:* PLGA with a ratio of 50/50 lactic acid to glycolic acid (Resomer RG505, inherent viscosity 0.73 dL·g^−1^, molecular weight of 54–69 kDa) was bought from Evonik Technology GmbH, Essen, Germany. Chitosan (medium molecular weight, 190–310 kDa), sodium alginate, anhydrous tetrahydrofuran (THF), ibuprofen (IBU), and benzyltriethylammonium chloride (BTEAC) were bought from Sigma-Aldrich (Sigma-Aldrich, Buchs, Switzerland) and used as received. *N,N*-dimethyl formamide (DMF) was purchased from Sigma-Aldrich (Sigma-Aldrich, Switzerland) and dried over molecular sieves (4Å) prior to use. All the water used in this study was Milli-Q^®^ water (Veolia, Basel, Switzerland).

*Preparation of PLGA electrospun nanofibers:* An in-house built electrospinning setup, as previously reported, was used for the fabrication of PLGA electrospun nanofibers [37]. PLGA was dissolved in a solvent system of 85/15% (*v*/*v*) THF/DMF to achieve a final polymer concentration of 15% (*w*/*v*). BTEAC was added to the solution at a concentration of 0.1% (*w*/*v*). A syringe equipped with a 0.456 mm (21G) diameter needle was filled with the polymer solution and loaded on a syringe pump. The polymer solution was emitted at a rate of 20 µL.min^−1^ under a potential difference of 20 kV (+15/−5 kV) with a needle-to-collector distance of 20 cm on a rotating drum (50 rpm) covered with aluminum foil. The membranes were electrospun for 4 h. Then, the mats were dried overnight in a vacuum oven at 40 °C. To assess the morphological properties of the electrospun nanofibers, the specimens were coated with 8 nm Au/Pd (EM ACE600, Leica Microsystems, Opfikon, Switzerland) and analyzed using an S-4800 scanning electron microscope (Hitachi High-Technologies, Schaumburg, IL, USA). The average diameter of the nanofibers was measured using ImageJ (ImageJ2, USA) [38]. For each specimen, n = 50 random measurements were performed on a total number for each picture per condition. (Figures S1 and S2).

*Plasma coating of PLGA electrospun nanofibers:* In order to introduce an oxygen-containing functional hydrocarbon layer onto the surface of the electrospun nanofibers, an ultrathin plasma coating (≤20 nm) was used as described in previous reports [39,40,41]. The coating comprises a vertical chemical gradient by changing the power and gas flow rate during the plasma deposition process in order to enhance adhesion, stability, and surface functionality [42]. Briefly, the electrospun nanofibers were introduced in a radio frequency-driven plasma chamber with a power input of 70 W, a pressure of 0.1 mbar, and exposed to a mixture of 8 sccm of carbon dioxide (CO_2_) with 4 sccm of ethylene (C_2_H_4_) for 190 s. Afterwards, the power was lowered to 30 W while the CO_2_ gas flow rate was increased to 24 sccm for another 50 s of plasma exposure. The plasma-coated meshes were kept under vacuum in a desiccator prior to further usage.

*Polyelectrolyte complex deposition* via *layer-by-layer assembly:* Then, plasma-coated electrospun nanofibers were coated with CHI and ALG. CHI was dissolved for 48 h in 0.8% (*v*/*v*) acetic acid to achieve a final concentration of 0.1% (*w*/*v*), while ALG was dissolved for 48 h in water at a concentration of 0.1% (*w*/*v*). Both solutions were adjusted to pH 5 with 0.1 M HCl. Nanofibrous meshes were cut into 50 mm diameter circles (≈100 mg) and placed on a Büchner filter membrane. Then, 50 mL of the CHI solution was suctioned through the mesh under vacuum for each side of the mesh. Afterwards, membranes were washed three times with 150 mL of MilliQ water on each side of the meshes to remove excess CHI from the surface. Then, a layer of ALG was added using the same procedure for the coating and washing. This process was repeated until a desired number of layers was obtained (5, 9, and 15). Finally, the membranes were dried at 40 °C in a vacuum oven. The coated nanofibers were kept under vacuum before further characterization. The meshes with 5, 9, or 15 layers are referred to as (CHI/ALG)_5_, (CHI/ALG)_9_, and (CHI/ALG)_15_.

*X-ray photoelectron spectroscopy:* A LS5600 X-ray photoelectron spectroscope (Physical Electronics, Chanhassen, MN, USA) was used to assess the chemical environment of the surface of the nanofibrous meshes. The spectrometer was equipped with a standard Al-Kα source, and the resolution was set to 0.8 eV.step^−1^ at a pass energy of 187.85 eV for survey scans and 0.25 eV at a pass energy of 46.15 eV for region scans. The acceleration voltage and current of the X-ray beam were set to 13 kV and 15 mA, respectively. Casa XPS (Casa Software Ltd., Teignmouth, UK) was used for data analysis, whereas carbon 1S was used for the calibration of the spectra at 284.5 eV.

*ζ-potential measurements:* The ζ-potential of the electrospun meshes was measured in response to the pH, in a range of 3 to 9, using a Surpass 3 electro-kinetic flow through a ζ-potential meter (Anton Paar, Graz, Austria). A pulsating stream of electrolyte solution (1 mM KCl in MilliQ water) was generated along the membrane to shear off the double layer, thus allowing the measurement of a streaming potential. The membranes were first rinsed 3 times with the electrolyte solution at the respective pH prior to the measurement.

*Infrared spectroscopy:* Attenuated Total Fourier Transform Infrared (AT-FTIR) spectra were recorded with a Varian 640-IR FTIR (Agilent Technologies, Santa Clara, CA, USA) on the sample surface. For each specimen, spectra were recorded at a range of 500 to 4000 cm^−1^ with a spectral resolution of 1 cm^−1^.

*Drug loading:* To study the kinetics of the loading from the electrospun constructs in response to pH, IBU was used as a model drug. For loading the drug, the meshes (5.4 ± 1.9 mg) with different numbers of polysaccharide layers were immersed in 2 mL of a saturated solution of IBU in MilliQ water (0.021 mg⋅mL^−1^) at pH 2.0, 5.0, 7.0, and 10.0. At specific time points (15 m, 30 m, 1 h, 2 h, 4 h, 6 h, 8 h, and 10 h), 30 µL of the solution was collected and replaced with 30 µL of fresh water solution of the same pH. Then, the collected supernatants were diluted to achieve a final volume of 500 µL, which is the minimum volume required for Ultra High-Performance Liquid Chromatography (UHPLC) measurements. To quantify the amount of drug entering the mesh, an Acquity UHPLC (Waters Inc., Baden, Switzerland) equipped with a UV-vis detector was used. Separation was achieved by reverse-phase gradient elution from 95/5% to 5/95% (*v*/*v*) water/acetonitrile over 5 min. The mobile phase was delivered at a flow rate of 0.5 mL⋅min^−1^ through an Acquity^®^ C18 column (2.1 × 50 mm, 1.7 µm). The detector wavelength was set at 221 nm, and the injection volume was 15 µL. Calibration curves using standard concentrations of IBU for the different pH values were recorded and used for quantification using the MassLinx^®^ interface. A typical chromatogram for IBU, the integration parameters, and the calibration curves for all the experiments can be found in Figures S3–S5 as well as Table S1. For further experiments, the specimens were immersed in a saturated solution of ibuprofen at pH 2.0 for 1 h and further washed two times with MilliQ water. The amount of drug lost during the washing steps was taken into account for further experiments.

*Release experiments:* After loading the meshes at the pH offering the highest loading capacity, the release of IBU was evaluated at 37 °C in phosphate-buffered saline (PBS) solution at pH 5.5 and 7.4 as well as in simulated gastric fluid (SGF, NaCl 2 g⋅L^−1^, pH 1.0). For this purpose, similar strips (6.0 ± 2.1 mg) of the loaded samples were immersed in 3 mL of each buffer solution. At specific time points (30 m, 1 h, 1 h 30 m, 2 h, 4 h, 12 h, 24 h, 48 h, and 72 h), 300 µL of the supernatant were collected and replaced with 300 µL of fresh buffer solution to keep the volume of the release media constant at 3 mL. Then, the collected supernatants were diluted to 500 µL and quantified using the aforementioned UHPLC method.

The release curves were fitted with a Ritger–Peppas model according to the equation:

(1) $$\frac{M_{t}}{M_{\infty }}=Kt^{n}$$

where $M_{\infty }$ is the amount of drug at the equilibrium state, $M_{t}$ is the amount of drug released over time (amount of drug loaded), *K* is the release velocity constant, and *n* is the exponent of release [43,44,45].

*Statistical analysis:* Results are displayed as mean ± standard deviation. Based on the small sample size, no normal distribution could be assumed. Therefore, statistical significance was assessed by a Kruskal–Wallis test for the groups followed by pairwise comparison with the Dunn test (Bonferroni corrections), and results were accepted as significantly different for *p* < 0.05. All the tests were performed in R, and the *p*-values mentioned in the text are the adjusted values [46].

## 3. Results

### 3.1. Preparation and Characterization of the Electrospun Nanofibers

#### 3.1.1. Electrospinning

**Figure 2:**
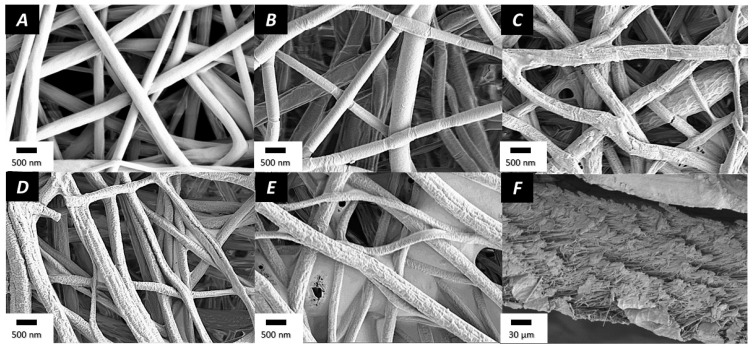
SEM pictures of (**A**) PLGA nanofibers, (**B**) plasma-coated nanofibers, (**C**) (CHI/ALG)_5_, (**D**) (CHI/ALG)_9_, (**E**) (CHI/ALG)_15_, and (**F**) cross-section of (CHI/ALG)_15_ layers.

PLGA electrospun nanofibers were successfully prepared. While the electrospinning of PLGA produced fibers in the micron scale, adding salt (BTEAC) to increase the conductivity of the solution resulted in fibers in the nanoscale range, with an average diameter of 547 ± 105 nm. The fibers were homogeneous and bead-free, as depicted in Figure 2A.

#### 3.1.2. Plasma Coating and Layer-by-Layer Assembly

To introduce negative charges over the surface of pristine PLGA electrospun nanofibers, the fibers were submitted to a radio-frequency plasma gradient to lower the charge of the mesh [47]. The morphology and the diameter of the fibers remained unchanged by the plasma coating, and the resulting nanofibers had an average diameter of 539 ± 118 nm (Figure 2B). After grafting the CHI/ALG polyelectrolyte complex, the surface of the electrospun fibers became granular (Figure 2). The average diameter of the fibers did not change significantly and was 550 ± 124 nm, 545 ± 109 nm, or 547 ± 137 nm for (CHI/ALG)_5_, (CHI/ALG)_9_, or (CHI/ALG)_15_, respectively (Figure 2C–E). The distribution of the diameters for 50 random measurements is shown in Figure S1. A cross-section of (CHI/ALG)_15_ was also performed to study the inner layers of the mesh (Figure 2F). Furthermore, lower magnifications of the meshes to study the homogeneity of the coating can be found in Figure S2 and revealed the poor homogeneity of the deposition of the coating.

#### 3.1.3. Fourier-Transform Infrared Spectroscopy

**Figure 3:**
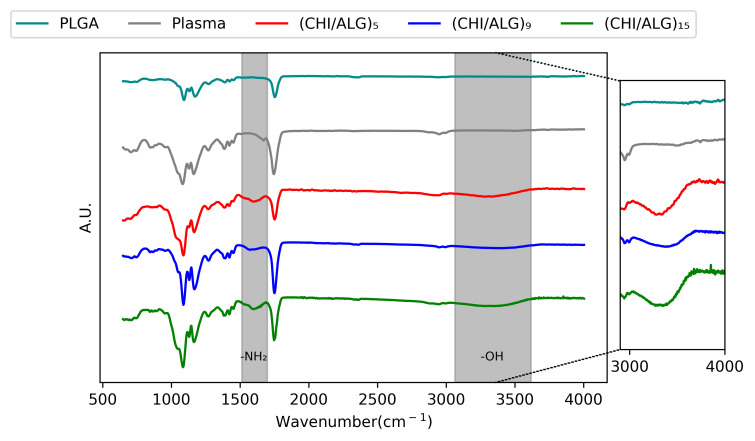
AT-FTIR of PLGA, plasma-coated, (CHI/ALG)_5_, (CHI/ALG)_9_, and (CHI/ALG)_15_ electrospun nanofibers. The inset highlights the differences between the peaks in the 3000–4000 cm^−1^ region corresponding to the alcohol function present on both alginate and chitosan polymeric backbones. The region from 1550 to 1650 cm^−1^ describes the apparition of the amino function present in the chitosan polymeric backbone.

AT-FTIR spectroscopy was used to confirm the presence of CHI on the surface of the nanofibers (Figure 3). For pure PLGA nanofibers, a strong peak is observed between 1760 and 1750 cm^−1^, which is characteristic for carbonyl groups (C=O). Another band is observed between 1300 and 1150 cm^−1^, corresponding to the ester (O=C-O) groups of PLGA. After LbL coating, a broad band appeared at 3500 cm^−1^, corresponding to the amine and hydroxyl groups of CHI. Another peak at 1623 cm^−1^ appeared, corresponding to the N-H of the amino function of CHI [27,48].

#### 3.1.4. X-Ray Photoelectron Spectroscopy (XPS)

**Figure 4:**
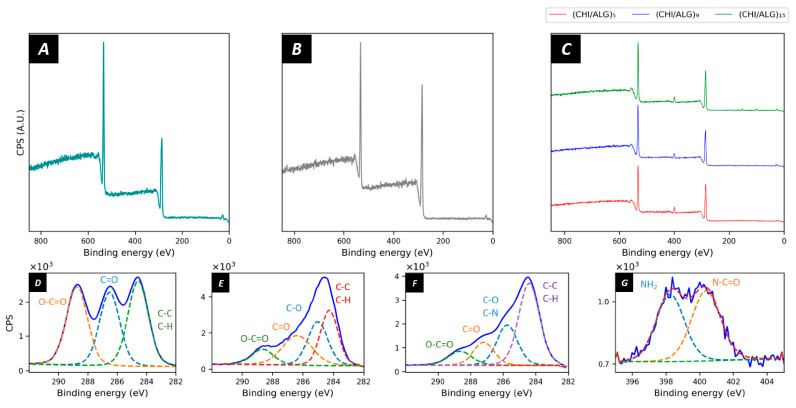
XPS survey scans of (**A**) PLGA, (**B**) plasma-coated PLGA, (**C**) (CHI/ALG)_5_, (CHI/ALG)_9_, and (CHI/ALG)_15_. The latter demonstrates a succesful deposition of chitosan due to the apparition of the typical peaks at 396–404 eV, depicting the presence of nitrogen onto the surface of the meshes. The region scans for (**D**) C1s of PLGA, (**E**) C1s of plasma-coated PLGA, and (**F**) C(1s) of (CHI/ALG)_5_ show the chemical changes on the surface throughout the different steps of the coating. The region scan for (**G**) N1s of (CHI/ALG)_5_ was used as an example of the nitrogen peak appearing after the deposition of chitosan.

X-ray photoelectron spectroscopy (XPS) was used to study the surface composition of the electrospun meshes (Figure 4). Pure PLGA nanofibers had the expected elemental composition as reported in the literature with the characteristic O1s peak at 533 eV and C1s peak at 284.5 eV, thus confirming the carbon and oxygen elements of the PLGA polymer matrix [49]. The elemental composition revealed 60.0% carbon and 39.5% oxygen at the surface of the meshes (Figure 4A). The chemical environment revealed the typical peaks of C1s corresponding to O=C–O (31.7%), C=O (33.3%) C–C, and C–H (35.0%) bonds at 288.3, 286.4, and 284.5 eV, respectively (Figure 4D). After plasma coating (Figure 4B), the amount of C1s on the surface of the nanofibers increased to 72.9% and the O1s decreased to 28.8%. When looking at the region scans (Figure 4E), the peak shape for C1s did not show the three characteristic peaks of PLGA anymore; however, a broader peak appeared. The peak fitting revealed the presence of C–O (32.0%), C=O (20.6%), O–C=O (12.1%), C–C, and C–H (35.4%) bonds. After the grafting of the CHI and ALG layers (Figure 4C), a peak appeared at 396–404 eV, revealing the deposition of nitrogen on the surface of the nanofibers. The nitrogen content at the surface of the membrane was 5.2%, 4.8%, or 5.2% for (CHI/ALG)_5_, (CHI/ALG)_9_, or (CHI/ALG)_15_, respectively. The spectra for ALG and CHI can be found in the Supporting Information (Figure S6).

#### 3.1.5. ζ-Potential Measurements

**Figure 5:**
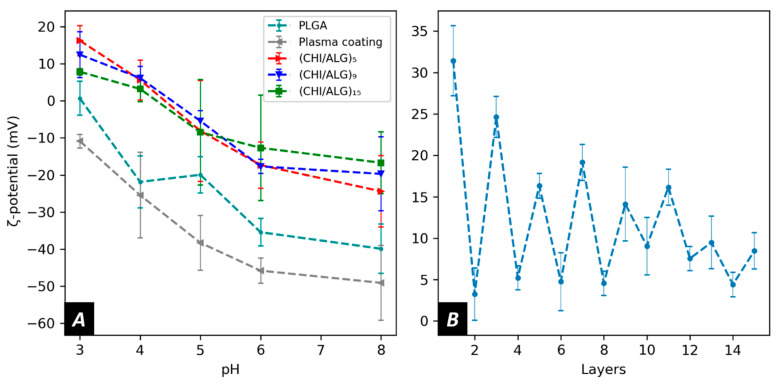
Surface zeta potential of (**A**) PLGA, plasma coated, (CHI/ALG)_5_, (CHI/ALG)_9_, (CHI/ALG)_15_, and (**B**) of the layer deposition process at pH 3.

To obtain a better understanding of the electrostatic forces involved during the LbL assembly, the surface ζ-potential at each step of the coating was measured. Pure PLGA nanofibers showed a negative ζ-potential over the range of pH measured (−20.0 ± 4.9 mV at pH5). After plasma coating, the ζ-potential of the membranes generally decreased to lower values (−38.3 ± 7.4 mV at pH 5). Furthermore, after the deposition of a first layer of CHI onto the surface, the ζ-potential shifted to positive values in acidic pH (Figure 5A), but after depositing a layer of ALG, the ζ-potential of the meshes returned to lower values (Figure 5B) while remaining positive.

### 3.2. Drug Loading and Drug Release from the Chitosan/Alginates Modified Fibers

#### 3.2.1. Drug Loading

**Figure 6:**
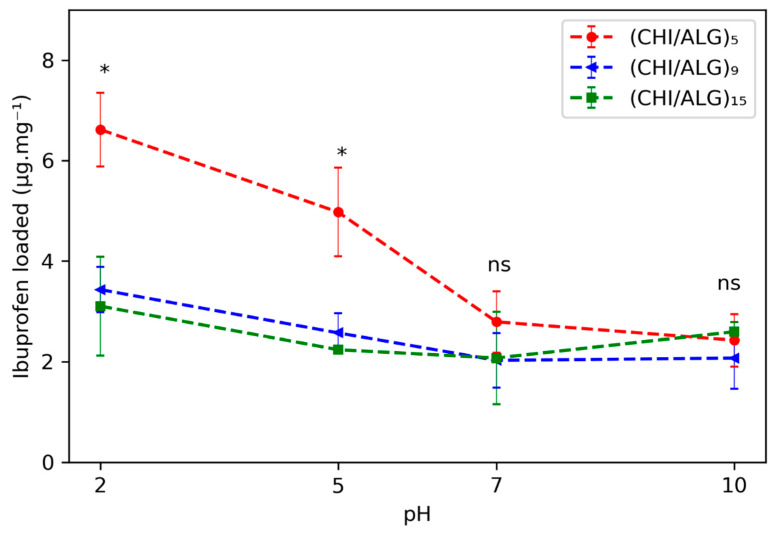
Amount of drug loaded onto the meshes pHs after 15 m of immersion in a saturated solution of IBU at pH 2.0, 5.0, 7.0, and 10.0. Statistical significance between (CHI/ALG)_5_ and (CHI/ALG)_9_ or (CHI/ALG)_15_ is highlighted with a * and non-significant differences between amounts are indicated with text (ns).

The loading kinetics of IBU within the different meshes were evaluated and were pH responsive. The amount of drug loaded was evaluated at different pH values (2.0, 5.0, 7.0, and 10.0; see Figure 6). The loading of the mesh was done by immersing the fibers in a saturated solution of IBU in the aforementioned pH range. The highest amount of drug loaded within most of the meshes was at pH 2.0, where 6.6 ± 0.7, 3.4 ± 0.4, and 3.1 ± 1.0 μg·mg^−1^ were loaded in the (CHI/ALG)_5_, (CHI/ALG)_9_, or (CHI/ALG)_15_ meshes, respectively. An increasing number of layers lowered the amount of drug incorporated within the mesh for pH 2.0 and 5.0 when comparing (CHI/ALG)_5_ with (CHI/ALG)_9_ and (CHI/ALG)_15_ (*p*-values < 0.05), while for higher pH, the amount of layer did not change the amount of drug loaded. The time-dependent loading curves are shown in Figure S7, and the drug penetrated the coating immediately, as the amount of drug loaded within the fibers did not change between 15 m and 24 h. The drug loading efficiency and the drug content within the fibers calculated using Equations S1 and S2 and are shown in Table S2. For subsequent release experiments, the drug was loaded by immersing the fibers in pH 2.0 for 1 h.

#### 3.2.2. Drug Release

**Figure 7:**
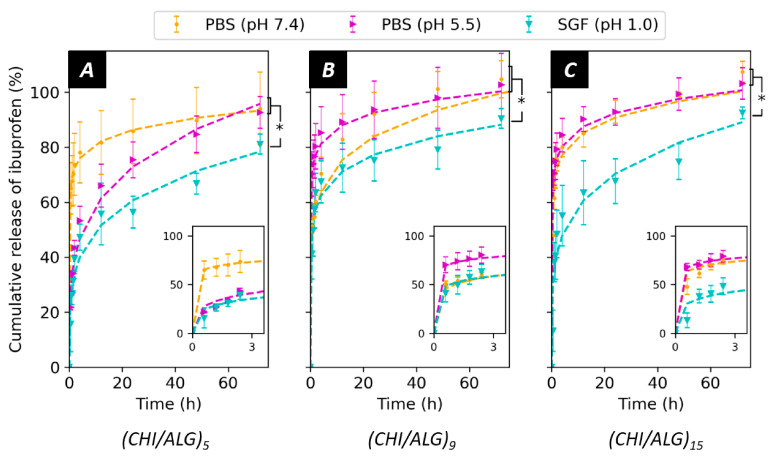
Drug release in PBS (pH 7.4), PBS adjusted to pH 5.5 and SGF (pH 1) from (**A**) (CHI/ALG)_5_, (**B**) (CHI/ALG)_9_, and (**C**) (CHI/ALG)_15_. The release rates were fitted using the Ritger–Peppas (power law) model. Statistical significance is indicated with * when comparing the release PBS (pH 7.4) and PBS (pH 5.0) with the release in SGF(pH 1.0).

The release of IBU from the meshes was measured in PBS at pH 7.4 and pH 5.5 as well as in SGF (pH 1.0, Figure 7). The release is depicted by a strong burst release, where most of the drug was released over 5 h, followed by a more sustained release over a 3-day time span. No significant differences in the release kinetics were observed when adjusting PBS from pH 7.4 to pH 5.5 (*p*-values > 0.1). Nevertheless, IBU was delivered slower in acidic pH (SGF, pH 1.0, *p*-values < 0.05) when compared to the other two pH environments. The insets show the release curves during the first three hours. The number of layers added to the surface did not affect the kinetics of the release. The curves were fitted using the Ritger–Peppas model, which provides precious information concerning the diffusion from the mesh, and the values for the model can be found in Table S2.

## 4. Discussion

Electrospinning of PLGA is well documented in the literature, and several studies show the impact of solvent and electrospinning conditions on the resulting nanofibers [50,51,52]. The fibers obtained by directly electrospinning PLGA dissolved in THF/DMF were in the microscale, while, to fully exploit the high surface-to-volume ratio of the mesh, fibers in the nanoscale were desired. Adding 0.01% (*w*/*v*) BTEAC to the polymer solution resulted in higher electrical conductivity, leading to nanoscopic fibers, as previously reported [53]. PLGA nanofibers exhibited a smooth surface and were bead-free, as shown in the SEM pictures (Figure 2). The grafting of CHI onto the surface of pure PLGA nanofibers proved to be unsuccessful. The negative surface charge of PLGA was insufficient to generate robust electrostatic interactions with CHI. To solve this issue, the nanofibers were submitted to a plasma gradient to lower the charge of the mesh as described in previous work [41].

Electrospun meshes are composed of several layers of fibers stacked on each other, forming a mesh that can reach several micrometers in thickness. The usual immersion coating on electrospun nanofibers is done by simply soaking the mat in a polymer solution. We found that by forcing the solution through the pores of the mesh using a Büchner filter directly connected to a high vacuum pump, a more homogeneous coating of the inner layers of nanofibers was achieved (data not shown here). This can be explained by a better interaction of the polymer solution with the different layers of the electrospun mesh as compared to simple immersion coating where the solution needs to penetrate through the mesh only via mechanical agitation. The rinsing steps were also performed using a Büchner filter to remove excess polysaccharides.

Using this approach, CHI and ALG were sequentially deposited onto the surface of the nanofibers to produce five, nine, and 15 layers, where the last layer was always CHI. ALG was used as a polyanionic entity to form the polyelectrolyte complex onto the surface and provided better cohesion throughout the coating. The surface morphology of the nanofibers appeared more granular after the LbL coating as described previously in the literature [29,36,54]. The pores appeared to be filled with polysaccharide after the deposition of nine and 15 layers, where the polyelectrolyte complexes covered the pores of the membrane, confirming previous findings [36,54]. This occurred throughout the whole membrane, as observed in the cross-section image (Figure 2F). A possible explanation could be the aggregation of the polyelectrolyte complex onto the surface throughout the coating steps or the splitting of the coated film into webs during the drying step. The lower magnification images in Figure S2 further show this phenomenon and the poor homogeneity of the coating on the meshes.

Further characterization using AT-FTIR confirmed the deposition of the polysaccharides on the surface of the electrospun nanofibers. The typical bands for the –NH bending vibration of the –NH_2_ amino group contained in CHI could be observed after the LbL assembly (Figure 1B). The penetration depth of infrared lying in the range of several micrometers confirms the successful deposition of the polysaccharides throughout the mesh.

Moreover, XPS was used to determine the surface composition of the electrospun nanofibers. The spectrum of PLGA exhibited the regular peaks for the polyester, and the peak fitting was in accordance with previous literature. After plasma coating, the shape of the carbon peak changed due to the incorporation of novel oxygen species on the surface. Furthermore, after coating the electrospun nanofibers via LbL, a clear peak appeared in the region of 396–404 eV. This peak can be attributed to the electrons emitted by the primary amines within the structure of CHI, and the nitrogen content appeared to be the same as the pure CHI powder (Figure S6). This validates that the CHI layers entirely cover the surface of the electrospun nanofibers.

To study the evolution of the surface charge during the coating, the ζ-potential of the meshes was measured using the streaming potential technique. The ζ-potential of pure PLGA electrospun nanofibers was negative over the measured pH range. This can be explained by the physical chemistry of polyesters, which have low pH isoelectric points, negatively charged ester groups, as well as negatively charged carboxylic end groups in their polymeric backbones. At higher pH, the ζ-potential of PLGA diminishes due to the dissociation of the carboxylic groups. After plasma treatment, the ζ-potential decreased, allowing the deposition of positively charged CHI onto the fibers.

The LbL assembly was performed at pH 5.0 to favor interaction between the dissolved polysaccharides as both CHI and ALG were charged at this pH. After depositing the first layer of CHI, the surface ζ-potential became positive in an acidic pH environment. The negatively charged carboxylic acid on alginate’s polymeric backbone led to a decrease in ζ-potential when ALG was added. The polyelectrolytes used for this study are oppositely charged, but no charge reversal was observed when adding ALG onto the surface. Such findings can be explained by the different charge densities of the polysaccharide used in our study [55]. Furthermore, as more layers were added, the change in surface charge became less evident (Figure 5B). This could be explained by the strong interactions within the polyelectrolyte complexes lowering the surface charge as most of the charges within the coating were screened by the oppositely charged polyelectrolyte.

After highlighting the presence of the polysaccharides on the surface of the electrospun nanofibers, we measured the capacity of the fibers to act as a drug delivery system by measuring their loading and release capacity. To evaluate the loading capacity of the coated electrospun meshes, we used UHPLC to follow the concentration of a saturated drug solution after immersing meshes of known weights. UHPLC provides an exceptional tool to detect IBU in solution, as the detection limit was accurate down to 20 ng.mL^−1^ and the separation over the column prevented any other compounds from interfering with the measurement. When studying the drug loading, more IBU penetrated the coating at pH 2.0 when compared to other pH values for most of the meshes (Figure 6). This can be explained by the protonation of the amino function of the CHI within the coating that allowed for better electrostatic interactions with IBU, which possesses a carboxylic acid in its structure.

It is noteworthy that the loading of the drug into the mesh was instantaneous, as the first time point was taken at 15 minutes, and the quantity of drug within the supernatant did not significantly change afterwards, regardless of the pH and the number of layers (Figure S7). We also found that (CHI/ALG)_9_ and (CHI/ALG)_15_ exhibited a lower amount of drug loaded at low pH (2.0 and 5.0) than (CHI/ALG)_5_, while the same amount of drug was loaded at higher pH (7.0 and 10.0) (Figure 6). As previously mentioned, when the number of polysaccharide layers stacked onto the surface increased, the electrostatic force from the resulting system diminished due to charge screening from the other layers. Therefore, the aforementioned interaction between CHI and IBU was disrupted as more layers were added, hence explaining the lower loading rates. Another phenomenon explaining the lower loading was the surface area diminishing with the increasing number of layers limiting the diffusion of drug molecules into the mesh. Due to the high amount of drug loaded at pH 2.0, this pH was used to load the drug and study the release from the mesh.

Alongside the drug loading, the release of IBU from the mesh was pH-sensitive. For all the measured samples, a strong burst release occurred, which can be associated with the desorption of IBU from the coating. In addition, the washing steps of the meshes in pH-neutral MilliQ water after the incorporation of the drug might trigger and initiate the release, leading to faster diffusion of the drug molecules to the surface. It is noteworthy to consider that the drug was assumed to be homogeneously distributed throughout the coating and that the coating was also assumed to be homogeneous when measuring the amount of drug loaded within the mesh and therefore released. Such assumptions and the low amount of sample measured leads to a difficult interpretation of the results and high standard deviations. This is also the explanation of values in the release profile exceeding 100%, as the amount of drug within the mesh could have been underestimated during the previous measurements.

After the burst release, significantly less drug was released from the mesh in acidic media (SGF, pH 1.0) than in PBS (pH 7.4 and 5.0). The pH-sensitive release can be explained by the existence of stronger interactions between IBU and the coating in an acidic environment, mainly between the protonated amino function of CHI and the carboxylic acid function of IBU. In addition, slower dissolution rates of IBU in acidic media could contribute to the observed kinetic rates [56]. When lowering the pH of PBS from 7.4 to 5.5, no significant changes could be observed in the release kinetics. This can be because not enough amino functions were protonated to interact and prevent the release of IBU. Furthermore, as suggested by the value of the release exponent (*n* < 0.5) of the Ritger–Peppas model, the release followed a diffusion-controlled mechanism [57,58]. The coefficient of determination values (>0.95, Table S2) also confirmed the validity of the model for the measured samples. Ibuprofen was chosen in this study as a model drug. Since the interactions between the coating and the drug regulate the release kinetics of the system, the use of other drugs will likely affect the release profile and offer different opportunities

Interestingly, the number of CHI/ALG layers onto the surface of the mesh did not affect the kinetics of the release. This was unexpected, as the ζ-potential of the surface and the drug loading were different when adding more layers and therefore should alter the interactions between IBU and CHI. Finally, the amount of IBU loaded and released by the measured meshes was in the therapeutic range of the drug (10–50 mg.L^−1^) [59]. In terms of applications, the pH-sensitive behavior of the meshes could prove to be a useful tool for the delivery of anti-inflammatory drugs required to pass through the digestive tracts known to have an acidic pH (1.0). Indeed, the proposed nanofibers could bypass the acidic environment of the stomach to allow for the delivery of therapeutics to the other part of the gastrointestinal tract [60]. For example, the jejunum or the ileum would be targets of choice for local delivery of ibuprofen using the fibers fabricated in this study.

## 5. Conclusions

In this study, we demonstrate the use of CHI/ALG coatings on electrospun nanofibers as a pH-sensitive drug delivery system. This simple and efficient way to decorate electrospun meshes via LbL paves the way to sophisticated functionalization in order to introduce novel properties to electrospun meshes. Here, we showed that the addition of CHI and ALG onto the surface led to the introduction of pH sensitivity, while the substrate (PLGA) is known to be inert toward changes in the environmental pH. While all materials used for this study have proven to be biocompatible in previous studies [61,62], further work involves the evaluation of the fabricated fibers in terms of biocompatibility. The grafting of materials onto the surface of electrospun fibers will be one of the major future directions for electrospinning as it allows for better biocompatibility, new functionalities, and the development of sophisticated systems.

## Data Availability

The raw data of this paper are fully accessible on request to the authors.

## Supplementary Materials

The following are available online at https://www.mdpi.com/article/10.3390/nano11071850/s1, Figure S1: Fiber distribution for (A) PLGA, (B) plasma coated, (C) (CHI/ALG)_5_, (D) (CHI/ALG)_9_, and (E) (CHI/ALG)_15_. Figure S2: SEM pictures showing the overview of (A) PLGA nanofibers, (B) plasma-coated nanofibers, (C) (CHI/ALG)_5_, (D) (CHI/ALG)_9_, and (E) (CHI/ALG)_15_. Figure S3: Typical chromatogram for ibuprofen (0.0105 mg/mL in PBS). Figure S4: Calibration curves of ibuprofen in water at (A) pH 2, (B) pH 5, (C) pH 7, and (D) pH10. Figure S5: Calibration curves of ibuprofen in PBS ((A) pH 7.4 and (B) pH 5.5 and in simulated gastric fluid (C) pH 1). Figure S6: XPS survey scan of (A) chitosan and (B) sodium alginate. Figure S7: Time-dependent loading curves at (A) pH 2, (B) pH 5, (C) pH 7, and (D) pH 10. Table S1: Apex Track Integration Parameters for ibuprofen. Table S2: Loading efficiency and the drug content within the fibers for the different pH of the different samples. Equation S1 and Equation S2 were used for the determination of the loading efficiency and the drug content, respectively. Table S3—Ritger–Peppas model parameters. Equation S1: Drug-loading efficiency formula. Equation S2: Drug content in the fiber formula
