# Supplementary material for: pH-Responsive Chitosan/Alginate Polyelectrolyte Complexes on Electrospun PLGA Nanofibers for Controlled Drug Release
Source: Nanomaterials (Basel). 2021 Jul 17;11(7):1850. doi: 10.3390/nano11071850 (PMC8308421; doi:10.3390/nano11071850)
Supplement: Supplementary file 1 [file nanomaterials-11-01850-s001.zip › nanomaterials-1264909-supplementary.pdf]

Supplementary Materials

pH-Responsive Chitosan/Alginate Polyelectrolyte Complexes on Electrospun PLGA Nanofibers for Controlled Drug Release

Jean Schoeller [1,2], Fabian Itel [1], Karin Wuertz-Kozak [3,4], Sandra Gaiser [5], Nicolas Luisier [1], Dirk Hegemann [5],
Stephen J. Ferguson [2], Giuseppino Fortunato [1,†] and René M. Rossi [1,2,*]

1. Empa, Swiss Federal Laboratories for Materials Science and Technology, Laboratory for Biomimetic Membranes and Textiles, 9014 St. Gallen, Switzerland; jean.schoeller@empa.ch (J.S.); fabian.itel@empa.ch (F.I.); nicolas.luisier@gmail.com (N.L.); giuseppino.fortunato@empa.ch (G.F.)
2. ETH Zürich, Institute for Biomechanics, 8093 Zürich, Switzerland; sferguson@ethz.ch
3. Schoen Clinic Munich Harlaching, Spine Center, Academic Teaching Hospital and Spine Research Institute of the Paracelsus Medical University Salzburg (AU), 81547 Munich, Germany; kwbme@rit.edu
4. Department of Biomedical Engineering, Rochester Institute of Technology (RIT), 14607 Rochester, NY, USA
5. Empa, Swiss Federal Laboratories for Materials Science and Technology, Laboratory for Advanced Fibers, 9014 St.Gallen, Switzerland; sandra.gaiser@empa.ch (S.G.); dirk.hegemann@empa.ch (D.H.)

* Correspondence: rene.rossi@empa.ch
† This author passed away in June 2020.

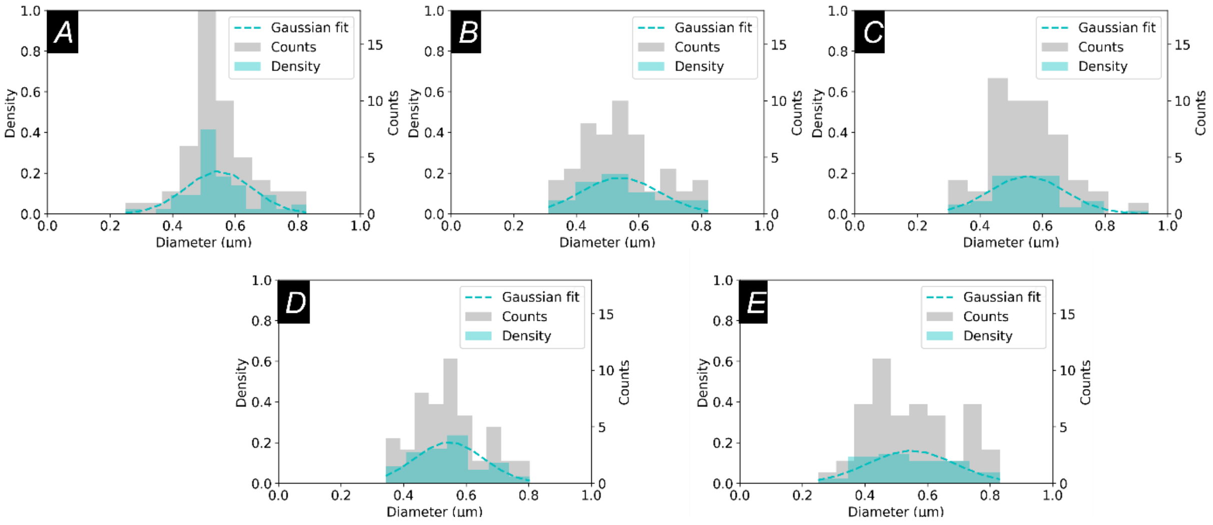

Figure S1: Fiber distribution for (A) PLGA, (B) plasma-coated, (C) $(CHI/ALG)_5$,(D) $(CHI/ALG)_9$ and (E) $(CHI/ALG)_{15}$. 5, 10 and 15 represent the number of layers of CHI and ALG deposited on the surface

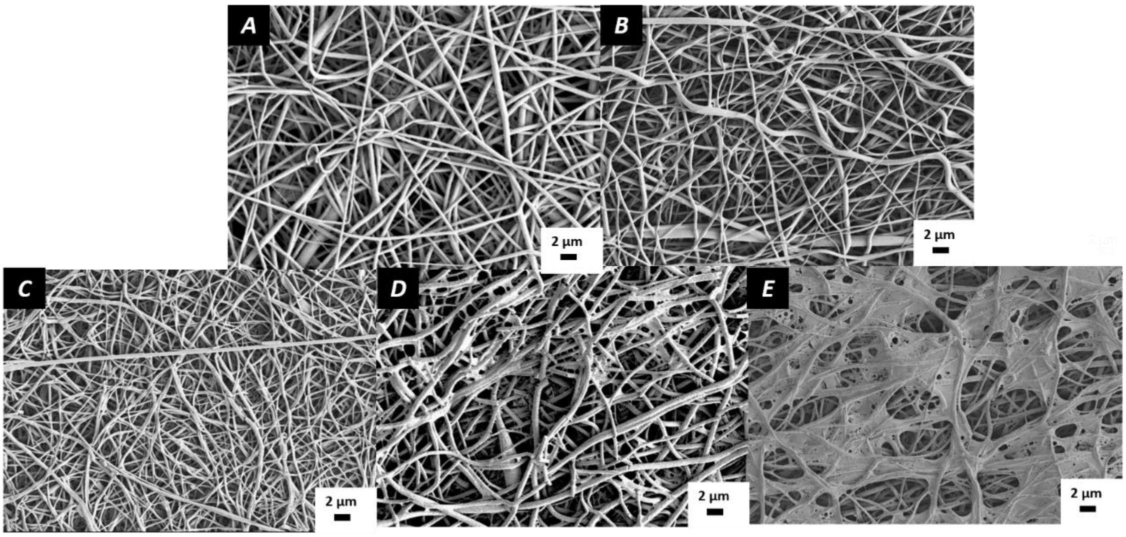

Figure S2 SEM pictures showing the overview of (A) PLGA nanofibers, (B) Plasma coated nanofibers, (C) $(CHI/ALG)_5$, (D) $(CHI/ALG)_9$ and (E) $(CHI/ALG)_{15}$

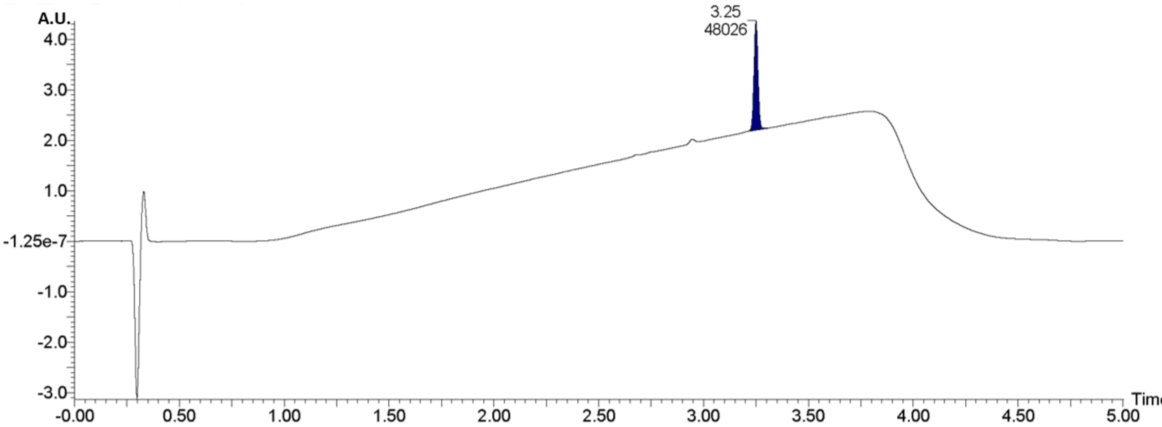

Figure S3: Typical chromatogram for ibuprofen (0.0105 mg/mL in PBS)

Table S1: Apex Track Integration Parameters for ibuprofen

**Retention time**	3.26 ± 0.04 minutes
**Peak-to-peak baseline noise**	44181.000
**Peak width at 5% height**	0.530
**Baseline start threshold %**	1.00
**Baseline end threshold %**	0.00

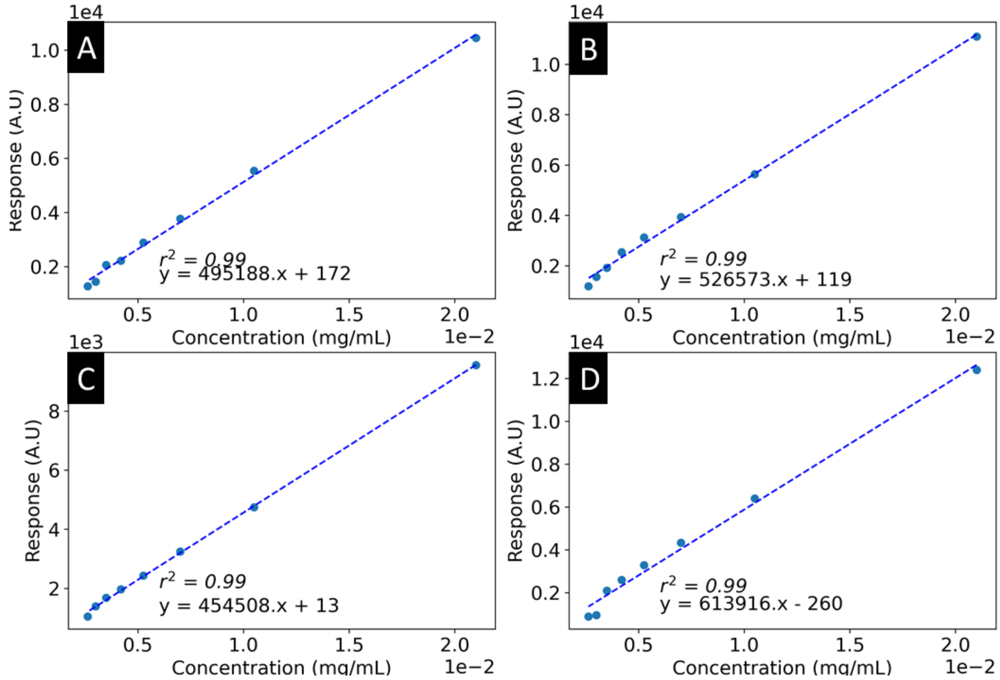

Figure S4: Calibration curves of ibuprofen in water at (A) pH 2.0, (B) pH 5.0, (C) pH 7.0, (D) pH10

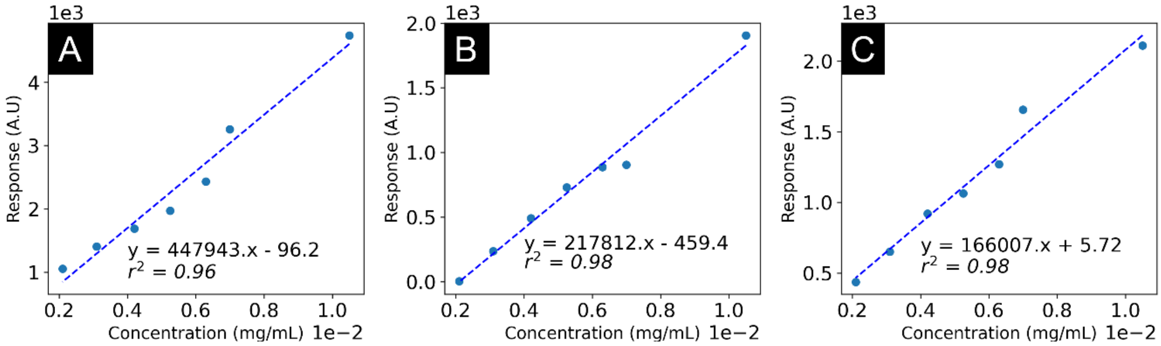

Figure S5: Calibration curves of ibuprofen in PBS ((A) pH 7.4 and (B) pH 5.5 and in simulated gastric fluid (C) pH 1.0)

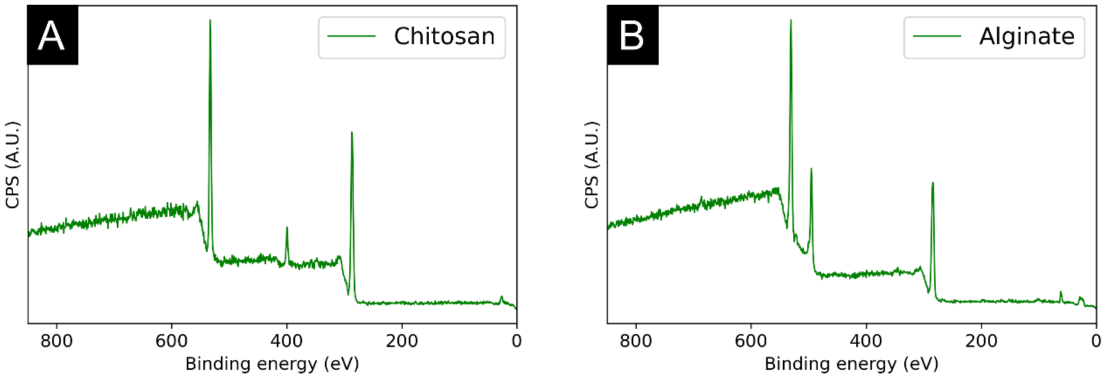

Figure S6: XPS survey scan of (A) chitosan and (B) sodium alginate

Equation S1: Drug loading efficiency formula

$$\textit{Loading efficiency}\ (\%) = \frac{\textit{Total mass of ibuprofen in saturated solution} - \textit{Mass of ibuprofen in the supernatant}}{\textit{Total mass of ibuprofen in saturated solution}}$$

Equation S2: Drug content in the fibers formula

$$\boldsymbol{Drug\ content}\ (‰) = \frac{\boldsymbol{Mass\ of\ ibuprofen\ loaded\ in\ the\ fibers}}{\boldsymbol{Mass\ of\ fibers}}$$

Table S2: Loading efficiency and the drug content within the fibers for the different pH of the different samples. Eq S1 and Eq S2 were used for the determination of the loading efficiency and the drug content, respectively.

Sample name	pH	Loading efficiency (%)	Drug content in the fibers (‰)
$(CHI/ALG)_5$	2.0	61.6 ± 2.6	6.6 ± 0.7
	5.0	40.5 ± 7.2	5.0 ± 0.9
	7.0	22.7 ± 6.0	2.8 ± 0.6
	10.0	33.3 ± 4.3	2.4 ± 0.5
$(CHI/ALG)_9$	2.0	59.9 ± 8.8	3.4 ± 0.5
	5.0	53.2 ± 5.4	2.6 ± 0.4
	7.0	33.5 ± 5.5	2.0 ± 0.5
	10.0	32.2 ± 2.0	2.1 ± 0.6
$(CHI/ALG)_{15}$	2.0	55.9 ± 9.4	3.1 ± 1.0
	5.0	48.7 ± 8.4	2.2 ± 0.1
	7.0	35.1 ± 13.8	2.1 ± 0.9
	10.0	28.3 ± 3.0	2.6 ± 0.2

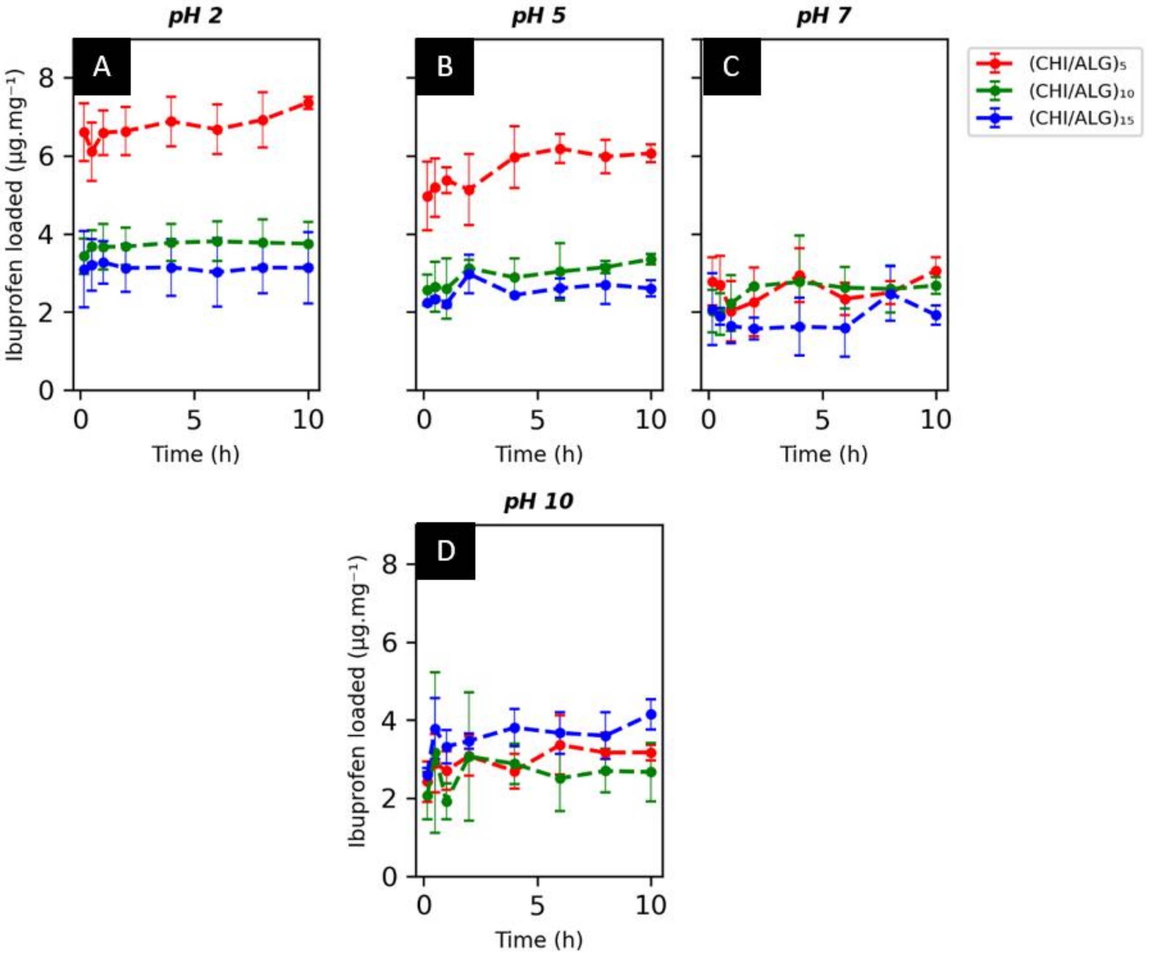

Figure S7: Time dependent loading curves at (A) pH 2, (B) pH 5, (C) pH7 and (D) pH 10. The amount of drug loaded remained the same throughout the time windowed. The amount of drug was quantified using UHPLC.

Table S3 – Ritger-Peppas model parameters for each speciments in the 3 measured pH (1.0, 5.5, 7.4)

		K	n	R^2
**$(CHI/ALG)_5$**	**PBS (pH 7.4)**	6.89	0.07	0.99
	**PBS (pH 5)**	34.98	0.24	0.97
	**SGF (pH 1.0)**	30.06	0.22	0.95
**$(CHI/ALG)_{10}$**	**PBS (pH 7.4)**	49.46	0.18	0.98
	**PBS (pH 5)**	76.52	0.07	0.99
	**SGF (pH 1.0)**	53.16	0.12	0.97
**$(CHI/ALG)_{15}$**	**PBS (pH 7.4)**	63.66	0.12	0.98
	**PBS (pH 5)**	72.97	0.08	0.99
	**SGF (pH 1.0)**	36.98	0.20	0.93
